# Keeping up with the information explosion: a surge in consumption of data on pediatric SARS-CoV-2 infection by pediatric emergency physicians

**DOI:** 10.1186/s12245-020-00285-x

**Published:** 2020-05-19

**Authors:** Oren Feldman, Amit Boukai, Itai Shavit

**Affiliations:** 1 Pediatric Emergency Department, Rambam Health Care Campus, Haifa, Israel; 2 Rappaport Faculty of Medicine, Technion-Israel Institute of Technology, Haifa, Israel; 3 Kibutz Maayan Tzvi, Israel

**Keywords:** SARS-CoV-2, Pandemic, Pediatrics

## Abstract

PEM-Database.org is an unaffiliated, not-for-profit website, dedicated to the field’s advancement of pediatric emergency medicine. PEM-Database published the first early access pediatric-related SARS-CoV-2 articles on March 13th, two days following the World Health Organization’s declaration of a global pandemic. Over the following 2 weeks, the number of PEM-Database entries increased dramatically. This surge expresses interest by pediatric emergency medicine physicians in data on pediatric SARS-CoV-2 infection.

To the Editor,

PEM-Database is a well-known online evidence-based resource for professionals practicing pediatric emergency medicine and is an unaffiliated, not-for-profit website, dedicated to the field’s advancement (http://www.pemdatabase.org/) [1]. PEM-Database is regularly updated with references to strictly academic and peer-reviewed material, such as pediatric emergency medicine abstracts, articles, and international guidelines published in the scientific literature, and is widely cited in leading pediatric and emergency medicine books [2–4]. The database is consistently edited and monitored by leading North American and European researchers [5]. The emergence of a coronavirus illness not previously seen in humans, called coronavirus disease 2019 (COVID-19), has captured the attention of the world. During December 2019, a series of pneumonia cases of an unknown origin were first identified in the city of Wuhan, Hubei, China. On December 31, 2019, China reported its outbreak to the World Health Organization, and shortly thereafter, the responsible pathogen was identified as a novel coronavirus, called SARS-CoV-2 due to its sequence similarity with the virus causing severe acute respiratory syndrome (SARS). By the end of February, a surge of cases spread throughout Europe and the USA, followed by an official declaration by the World Health Organization of a global pandemic on March 11, 2020. By March 28th, more than 645,000 cases of COVID-19 had been reported in over 190 countries and territories, with approximately 29,900 deaths [6].

PEM-Database published the first early access pediatric-related COVID-19 articles on March 13th, 2 days following the World Health Organization’s declaration. Until then, the website had a stable entry log ranging from 40 to 80 entries per day. *Yet, over the following 14 days, the number of entries increased to a maximum of 1193 entries per day, with an average 1240% higher than the previous week (Fig.**1**).* Nearly 45% of the site’s recent visitors were from the USA alone, and a combined 14% of entries were by physicians from Italy, Spain, France, and Canada, all of which are countries facing an outbreak. We believe that there is no other possible explanation for this surge other than a clearly specific interest by pediatric emergency medicine physicians in data on COVID-19 in children. Articles published on PEM-Database.org are strictly pediatric related; therefore, this increase in entries does not reflect a general interest in COVID-19 or in information about the illness in adults. This observed spike in entries to PEM-Database suggests that there is a great need and interest for up-to-date information on pediatric presentations of SARS-CoV-2 among pediatric emergency medicine physicians.

**Fig. 1:**
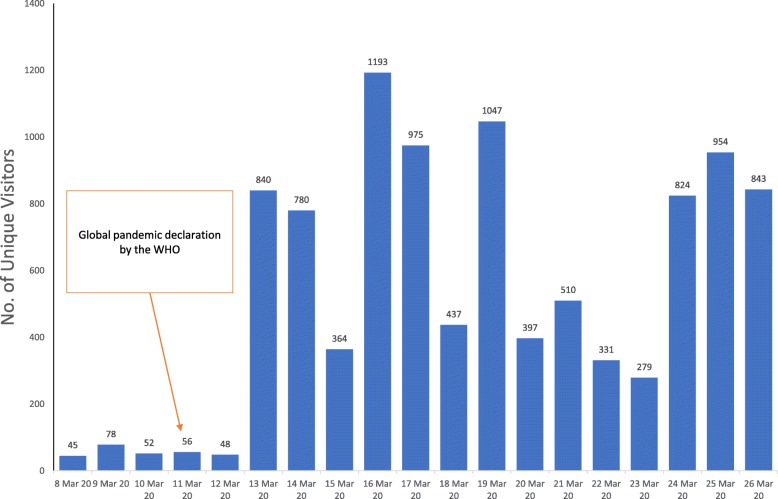
Number of entries to PEM-Database.org between March 8, 2020, and March 26, 2020

## Data Availability

All data is included in the paper and table.

## Abbreviations

COVID-19: Coronavirus disease 2019
SARS: Severe acute respiratory syndrome
